# Cardio-visual full body illusion alters bodily self-consciousness and tactile processing in somatosensory cortex

**DOI:** 10.1038/s41598-018-27698-2

**Published:** 2018-06-18

**Authors:** Lukas Heydrich, Jane Elizabeth Aspell, Guillaume Marillier, Tom Lavanchy, Bruno Herbelin, Olaf Blanke

**Affiliations:** 1 Laboratory of Cognitive Neuroscience, Brain Mind Institute, Ecole Polytechnique Fédérale de Lausanne, 1015 Lausanne, Switzerland; 2 Department of Neurology, Inselspital, Bern University Hospital, University of Bern, Bern, Switzerland; 3 Center for Neuroprosthetics, School of Life Sciences, Ecole Polytechnique Fédérale de Lausanne, 1015 Lausanne, Switzerland; 4 Department of Psychology, Anglia Ruskin University, Cambridge, UK

## Abstract

Prominent theories highlight the importance of bodily perception for self-consciousness, but it is currently not known whether this is based on interoceptive or exteroceptive signals or on integrated signals from these anatomically distinct systems, nor where in the brain such integration might occur. To investigate this, we measured brain activity during the recently described ‘cardio-visual full body illusion’ which combines interoceptive and exteroceptive signals, by providing participants with visual exteroceptive information about their heartbeat in the form of a periodically illuminated silhouette outlining a video image of the participant’s body and flashing in synchrony with their heartbeat. We found, as also reported previously, that synchronous cardio-visual signals increased self-identification with the virtual body. Here we further investigated whether experimental changes in self-consciousness during this illusion are accompanied by activity changes in somatosensory cortex by recording somatosensory evoked potentials (SEPs). We show that a late somatosensory evoked potential component (P45) reflects the illusory self-identification with a virtual body. These data demonstrate that interoceptive and exteroceptive signals can be combined to modulate activity in parietal somatosensory cortex.

## Introduction

Evidence for the role of multisensory integration in bodily self-consciousness is based on studies of both body-part^1–3^ and full body illusions^4–7^ which induce multisensory conflicts between external (‘exteroceptive’) sensory modalities, such as vision and touch. There is, however, growing support for the theory that the brain’s representations of internal (‘interoceptive’) bodily states (e.g., the heartbeat^8–12^) are equally or even more important for the self^13,14^, given their role in homeostatic, life-sustaining processes.

A full neurobiological account of bodily self-consciousness will need to explain how and where interoceptive and exteroceptive sources of bodily information are combined in the brain to form a global self-representation. Although we have some understanding of the convergence of visceral and somatosensory signals in single neurons in the spinal cord, brain stem, and thalamus^15–17^, little is known of the cortical involvement in intero-exteroceptive integration. This research is, as yet, in early stages, but there is evidence from clinical work^18^, neuroimaging studies^10,19–21^ as well as behavioural work suggesting interactions between interoceptive and exteroceptive processing on a cortical level^9,22,23^.

Relations between interoception and exteroception and the importance of their interactions for bodily self-consciousness has only very recently been studied. A paper by our research group^24^ was one of the first to look at these interactions in the context of a full body illusion (see details below and^25^). Park and colleagues more recently showed^21^ that the amplitude of a cortical index of interoception (the heartbeat evoked potential) predicts the conscious visual detection of a barely perceptible stimulus. Links between visual and cardiac processing were also highlighted by a study from our group showing that activation in the insula (a key interoceptive cortical area) was suppressed when a visual stimulus was presented in synchrony to the participant’s heartbeat^10^. Most studies so far^26^ have shown effects of interoception on exteroception; only one study, to our knowledge has shown the reverse: Marshall *et al*.^27^ found that repeating angry facial stimuli suppresses HEP amplitude.

At the same time, the role of atypical interoception in psychiatric (e.g. schizophrenia^28^) and developmental disorders (autism) has also received growing attention. Atypical sensory processing is one of the diagnostic criteria of autism^29^ and the finding that the severity of abnormal sensitivity correlates across different modalities suggests that individuals with autism have a global deficit in the processing of exteroceptive stimuli^30^. Most research on autism to date has focussed on exteroceptive sensory difficulties^31,32^; atypical interoception has been investigated only more recently. These latter studies have shown that interoceptive sensitivity is lower in people with autism^33^ and this is strongly linked to the degree of alexithymia^34–36^. These findings have led to the suggestion^37^ that the social difficulties of autism arise from malfunctions in the ‘oxytocin-interoception system’. The social difficulties have also been linked to the well-documented abnormalities in self processing reported in autism^38–41^. Atypical self-processing may be related to aberrant exteroceptive-interoceptive integration in people with autism^42^. Growing evidence therefore suggests that atypical social cognition, interoception and self-consciousness are likely co-dependent, inter-related characteristics of autism.

In a recent study^43^ we sought to explicitly test links between interoception and bodily self-consciousness by devising a new illusion in which interoceptive signals are ‘projected onto’ the participant’s virtual body. In particular, we investigated whether a conflict between an interoceptive signal (the heartbeat) and an exteroceptive (visual) signal would modulate bodily self-consciousness, and further, whether this cardio-visual conflict would also alter exteroception (tactile perception). We presented cardio-visual illumination of the virtual body so that a flashing silhouette was either temporally synchronous or asynchronous with respect to the participant’s heartbeats. Our data showed that participants self-identified more with the virtual body, and their self-location was biased more towards it in the synchronous compared to the asynchronous condition. Synchronous cardio-visual signals also altered the perception of tactile stimuli presented during the illusion.

In the present study we investigated whether the experimentally-induced changes in bodily self-consciousness induced by the cardio-visual illusion are accompanied by changes in somatosensory activity by recording somatosensory evoked potentials (SEPs) to medial nerve stimulations during the heartbeat illusion. Such information would give some insight into where in the brain a convergence of interoceptive and exteroceptive signals might occur. Based on our previous behavioural evidence for changes in tactile perception during the illusion, as well as the finding of both early and late SEP changes during the visuo-tactile full body illusion^44^, we expected to find an illusion-induced modulation of SEPs. Whereas Bufalari and colleagues^45^ reported that viewing painful tactile stimuli delivered to another’s body modulates the amplitude of the P45 to median nerve stimulation, Dieguez *et al*.^46^ showed that the N20 is enhanced during a body part ownership illusion. Given the non-somatosensory nature of our illusion, we predicted that multimodal areas (such as the secondary somatosensory cortex, the posterior part of the insular cortex or the posterior parietal cortex) rather than primary somatosensory cortex will be involved, and that this will be reflected by the P45 component (vs. the N20), as suggested previously^45^. This would also be in line with a recent observation by Ronchi and colleagues^9^ who reported a change in responses to the heartbeat illusion in a patient with a right insular lesion.

## Results

### Self-identification

**Figure 1:**
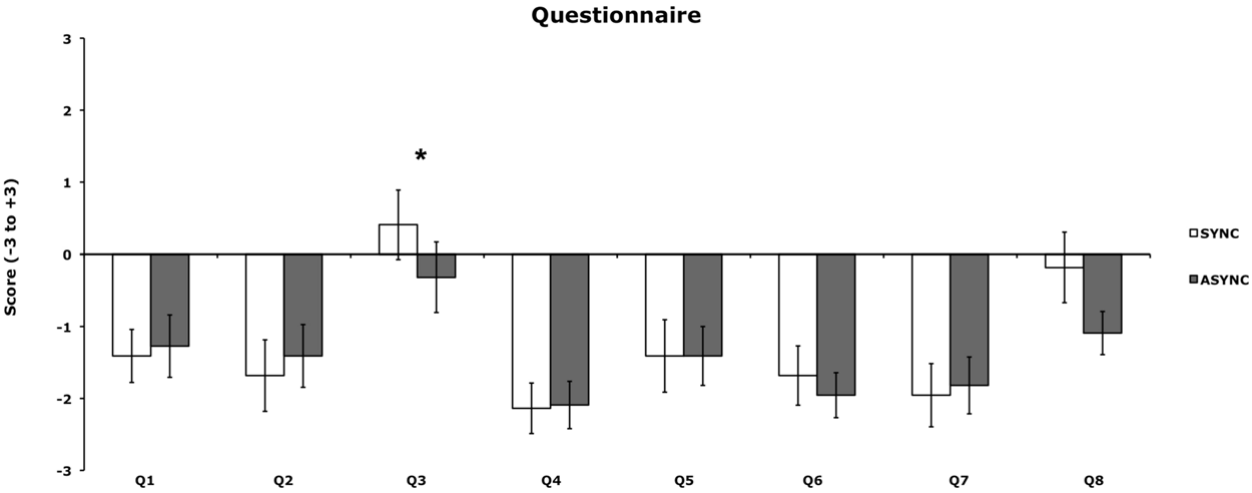
Behavioural results. Self-identification with the virtual body (Q3) was significantly modulated by cardio-visual synchrony. White bars indicate ratings in the synchronous condition (body synchronous condition; BS). Dark grey bars indicate ratings in the asynchronous condition (body asynchronous condition, BAS). Asterisks indicate significant differences. Error bars indicate standard error of the mean (SEM).

Analysis of questionnaire scores revealed that during the SEP recording participants self-identified more strongly with the virtual body in the BS condition (Q3: ‘I felt as if the body was my body’; mean = 0.41) as compared to the BAS condition (mean = −0.32, p = 0.0059, one-tailed). None of the other contrasts reached significance (see Fig. 1).

### Intensity ratings

No significant difference was found between the intensity ratings for BS (3.1 ± 2.3) and BAS (3.7 ± 2.3), BS and BL (2.8 ± 2), or BAS and BL (all p > 0.62; see Supplemental Fig. 1).

### SEP data

Three subjects did not show the classical components and were not included in further ERP analysis. The grand average SEP revealed classic SEP components peaking at N20, P25, N35, P45, and N55 at scalp electrode C3. Across all conditions the maximal peak amplitude for the N20 component was on average at 19.9 ± 1.4 ms. The maximal peak for the P45 component was at 45.9 ± 3.4 ms. A two-tailed paired t-test was applied to the latency data of each of the five SEP components, revealing no significant difference between the two conditions for any component (N = 10, all p > 0.26, see Supplemental Table 2). Furthermore, no significant difference was found between BS and BL (N = 10, all p > 0.07) and BAS and BL (N = 10, all p > 0.08).

**Figure 2:**
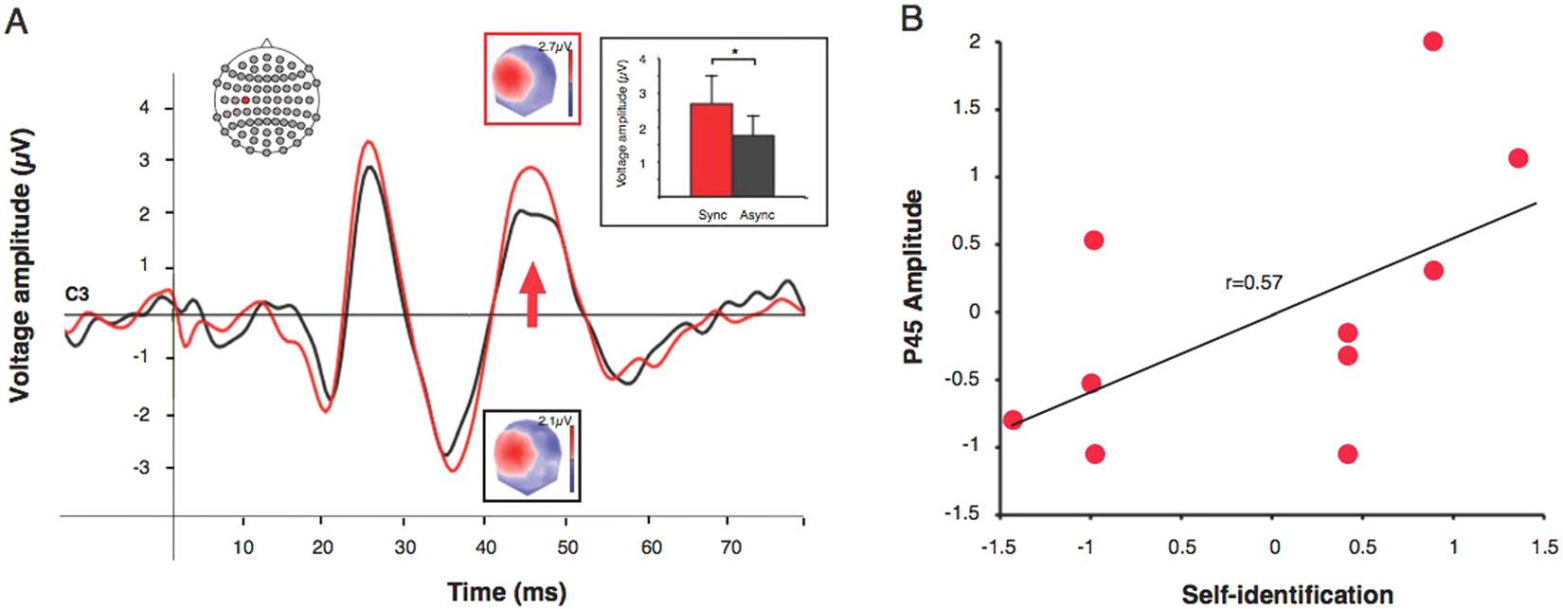
(**A**) Somatosensory evoked potentials. During the illusion condition (BS), only the amplitude of the P45 component of somatosensory evoked potentials (recorded at electrode C3 contralateral to the stimulated right median nerve, N = 10) was found to significantly differ from the control condition (BAS). The amplitude of the P45 component (grand average somatosensory evoked potential) in the two experimental conditions is shown in the small inserted plot (same colour-code as in the larger plot; red: synchronous; black: asynchronous; error bars are SEM). (**B**) Correlation. The plot shows the P45 amplitude relative to baseline at electrode C3 for each participant (N = 10). Analysis revealed that the amplitude of the P45 component correlated significantly and positively with the self-identification ratings with the virtual body. This was only found during the BS condition (amplitude and rating are plotted as standardized z-scores).

Analysis of the SEP data at electrode C3 (over somatosensory cortex contralateral to the electrically stimulated right median nerve) and C4 (ipsilateral to the stimulated nerve) revealed a significant 3-way interaction between electrode, synchrony, and component (F_1,9_ = 11.7, p = 0.009). Post-hoc analysis showed a synchrony-dependent modulation was only found at C3 and at 45 ms after stimulus onset (P45 component). Thus, only at C3 (and not at C4) a significant modulation of the SEP amplitude was found and this only for the P45 component (Fig. 2A; the P45 amplitude at C3 differed between the BS (2.52 +/− 0.66 μV) and BAS (1.71 +/− 0.59 μV) condition (p = 0.001) and was larger in the BS condition. No significant differences were found for the N20 component between BS (−1.65 +/− 0.38 μV) and BAS (−1.29 +/− 0.32 μV) at C3. No differences were found for either of the two components at electrode C4 (all p > 0.05, see Supplemental Fig. 2).

### Correlation

Further analysis showed that the P45 amplitude at electrode C3 correlated positively with self-identification ratings during the BS condition (Fig. 2B; N = 10, R = 0.61; p = 0.05). No significant correlation was found between the amplitude at electrode C3 and Q3 in the BAS condition nor between the amplitude of the N20 component at electrode C3 and Q3 for the BS and BAS condition (all p > 0.27).

### Source localization

**Figure 3:**
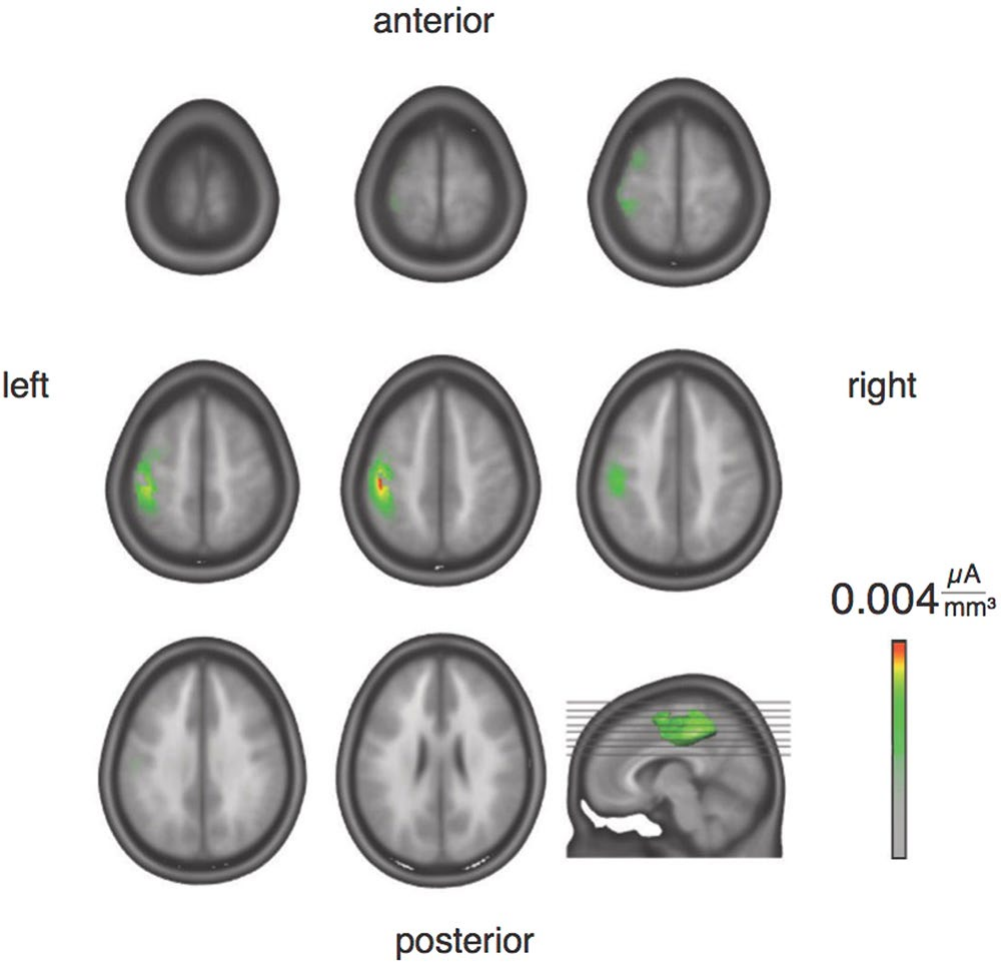
Source localization. Distributed linear inverse solution based on a local auto-regressive average (LAURA) model applied to the SEP component at P45 (after right median nerve stimulation) localized activity to left parietal cortex including the postcentral gyrus and extending to the inferior parietal cortex.

The application of a distributed linear inverse solution based on a local auto-regressive average (LAURA) model localized the P45 component to left contralateral parietal cortex ranging from the postcentral gyrus (Brodmann area 2, Talairach coordinates −47, −20, 47; strongest activation) and extending to the inferior parietal cortex (Brodmann area 40, Talairach coordinates −41, −44, 48; Fig. 3).

## Discussion

Our data are compatible with recent evidence pointing to the importance of exteroceptive *and* interoceptive systems for self-consciousness^10,18,25,42,43^. We show that signals from these systems – cardiac and visual - are associated with changes in self-identification and somatosensory processing. The present subjective data replicate those of a previous study from our group^43^, demonstrating that participants show stronger self-identification when an illuminating silhouette surrounding a video image of the participant’s own body flashed on and off synchronously with the participant’s heartbeat as compared to a control condition. This data adds further evidence to the notion of a multisensory integration model of bodily self-consciousness, e.g. that mechanisms for detecting correlations between the timing of an exteroceptive (flashing visual stimulus) *and* an interoceptive signal (heartbeat) contribute to the basis of bodily self-consciousness.

Based on our previous behavioural evidence for changes in tactile perception during the illusion^43^, we sought to investigate the associated brain mechanisms by measuring activity in somatosensory cortex during the illusion. We found a significant positive correlation between the amplitude of the P45 component and the strength of the illusion in the synchronous condition after right median nerve stimulation, providing preliminary electrophysiological evidence for a modulation of somatosensory activity during the cardio-visual illusion. Our finding of enhanced SEP amplitude in the synchronous condition is in line with a recent visuo-tactile full body illusion study from our lab^44^ which found that SEPs at 30–50 ms after tibial nerve stimulation were enhanced during the synchronous condition. SEPs recorded during a ‘numb finger’ illusion were also enhanced during the illusion condition^46^. All of these body illusion SEP findings are possibly consequences of a ‘functional deafferentation’ effect caused by ‘disownership’ of the body/a body part. This idea is based on the finding that physical deafferentation (e.g. anesthesia/nerve block), which has also been associated with loss of ownership^47^, leads to SEP enhancement^48,49^. Furthermore, it has been suggested that ownership illusions alter homeostatic and somatosensory processing of the physical body, as observed in the RHI, when the stroked hand becomes cooler^50^, in the FBI when the whole body cools^51^, and in the FBI when both acute and chronic pain sensations are reduced^52,53^.

Here, changes in bodily self-consciousness were reflected by the late (P45) SEP component but not by the early SEP component over the left hemisphere. This modulation of the late P45 SEP component (as opposed to earlier SEP components such as the N20 component that reflects activation restricted to S1) suggests that areas beyond S1 (such as the secondary motor cortex or the posterior insula^45,54,55^ are associated with changes in somatosensory processing linked to altered states of bodily self-consciousness (e.g. illusory self-identification with an avatar). This is supported by our finding that the application of a distributed linear inverse solution localized the P45 component to left contralateral parietal cortex ranging from the postcentral gyrus and extending to the inferior parietal cortex. Importantly, the observation that the amplitude of the P45 component correlated positively with the strength of self-identification with the virtual body in the illusion-inducing condition only, further supports the conclusion that the magnitude of this somatosensory activation induced by median nerve stimulation reflects changes in bodily self-consciousness, due to convergence of interoceptive, visual and somatosensory processing.

Viscero-somatic convergence has been described in the dorsal horn of the spinal cord, the brain stem and the thalamus by revealing single neurons with tactile receptive fields that also receive afferent cardiac input^15,17^, e.g. afferent signals from the viscera converge with somatosensory afferents from specific body parts^15^. Recently, Gray and colleagues showed an integration of somatosensory and phasic baroreceptor information at cortical, limbic and brainstem levels^19^. Abnormalities in the timing of the integration of interoceptive and exteroceptive stimuli found in autism^42^ may underlie the atypical sense of self which is characteristic of the disorder^38^. Others have found that visual processing and visuo-tactile integration, respectively is dependent on visceral afferent information (e.g. the heartbeat^10,21,56^) and have linked this process to the multisensory cortical areas, such as the insula^10,12^ and the inferior parietal lobule^21^. Importantly, in two very recent studies the same heartbeat illusion paradigm was used. Ronchi *et al*. tested a patient before and after resection of a right insula neoplastic lesion^9^.While before surgery the patient experienced self-identification with the virtual body in both the synchronous and asynchronous conditions, the illusion was only present in the synchronous condition after surgery. Blefari *et al*.^57^ demonstrated that activity in the bilateral Rolandic operculum showed the highest selectivity for bodily self-consciousness based on our heartbeat illusion. This provides further evidence that the insula and adjacent structures support the cardio-visual effects on bodily self-consciousness observed during the illusion. In our study the source localization of the P45 component did not highlight the insular cortex, but rather late somatosensory processing in secondary somatosensory cortex (SII, inferior parietal cortex). This is most likely due to the fact that we were measuring somatosensory signals time-locked to median nerve stimulation and its modulation by a cardio-visual illusion, and not responses to the cardio-visual illusion *per se*. Thus, the observed changes in somatosensory cortex may occur as a consequence of signals arriving from different brain regions such as the insula or the parietal cortex (e.g. posterior operculum). Cardio-visual signals could also have been integrated in the insula first and these integrated signals could then have modulated somatosensory processing and bodily self-consciousness.

The insula has been shown to be recruited during the perception of cardiac signals^58,59^ and has been proposed to be key area for interoception and subjective bodily feelings^60^. It is a highly multisensory brain region that is activated by visual, tactile, and auditory cues^61–63^ and it has been suggested that the posterior insular cortex^64^ combines somatosensory signals from the second somatosensory area (SII) with signals from limbic structures^65^, e.g. supporting the experience of affective touch (e.g. pleasant vs. neutral touch). The insula has been implicated in a number of studies on self-attribution of a fake or virtual hand^1,66^ as well as in illusory own body perceptions of neurological origin^18^ and disownership of body parts^67^.

Although attentional mechanisms have been shown to enhance SEP components^68,69^ and cannot be excluded in the present study, an attention explanation seems unlikely given (1) that participants were not aware of the cardio-visual synchrony, (2) attention would be expected to enhance several SEP components (not only the P45^68,69^), and also given (3), our finding that the strength of the P45 component correlated with self-identification ratings.

A limitation of the current study is the relatively low number of participants, which can result in an overestimation of the observed effect^70^. A replication of our findings in a higher powered sample is therefore desirable to test the robustness of this effect. However, the present data are in line with the behavioural results of a previous study from our group using the same paradigm^43^ and an independent research group using a similar setup based on the rubber hand illusion^25^. We therefore consider our findings valid and reliable.

In conclusion, the present changes in SEPs and self-identification during cardio-visual stimulation support the notion of a multisensory integration model of bodily self-consciousness, e.g. that mechanisms for detecting correlations between the timing of an exteroceptive (flashing visual stimulus) *and* an interoceptive (heartbeat) signal contribute to the neural bases of bodily self-consciousness, and modulate somatosensory processing.

## Methods

### Participants

A total of 13 healthy right-handed participants took part in the study (5 females, mean age 21.0 ± 2.4 years). All participants had no previous experience with the task or related experimental paradigms. All participants had normal or corrected to normal vision and had no history of neurological or psychiatric conditions. All participants gave written informed consent and were compensated for their participation. The study protocol was approved by the local ethics research committee – La Commission d’éthique de la recherche Clinique de la Faculté de Biologie et de Médecine – at the University of Lausanne, Switzerland and was performed in accordance with the ethical standards laid down in the Declaration of Helsinki.

### Materials and procedure

#### Setup, electrocardiogram (ECG), signal analysis

**Figure 4:**
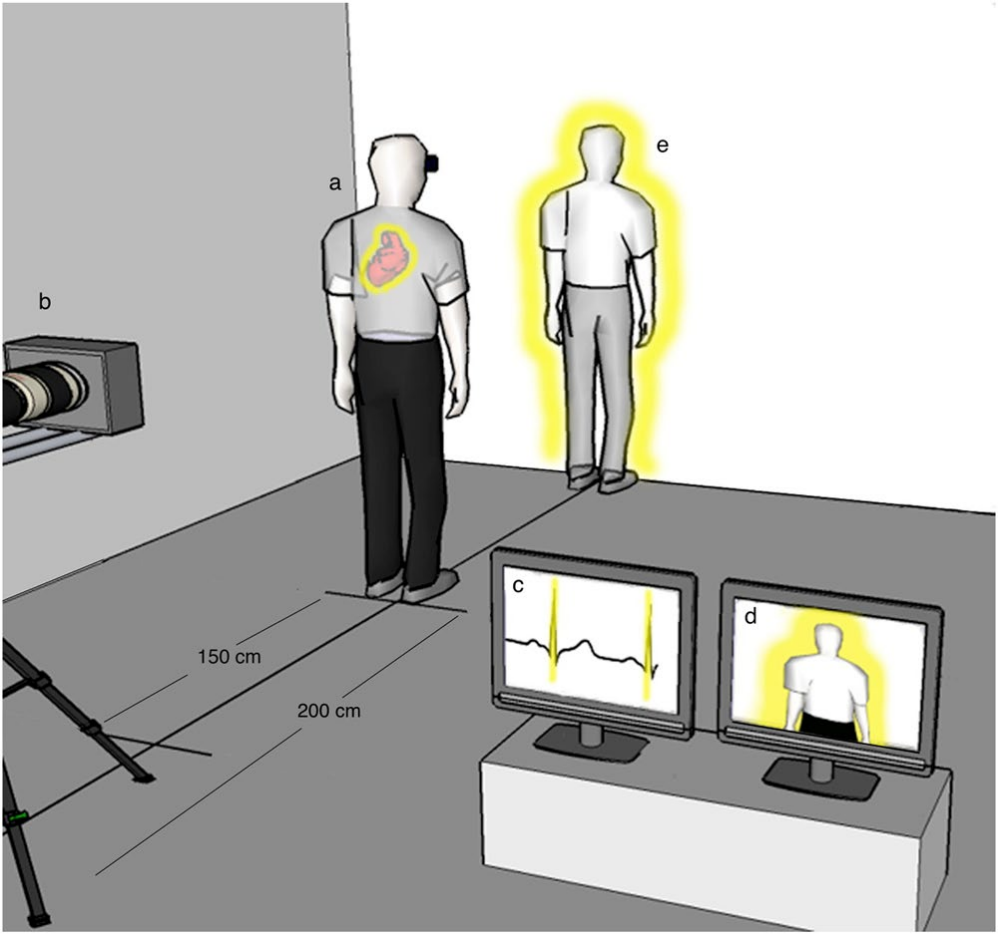
Setup. Participants (**a**) stood with their backs facing a video camera placed 200 cm behind them (**b**). An electrocardiogram was recorded (**a**) and R-Peaks were detected in real-time (**c**), triggering a flashing silhouette outlining the participant’s body (virtual body) (**d**). The video, showing the virtual body was projected in real time onto a head mounted display (HMD) (*body condition)*. It appeared visually that the virtual body was standing 200 cm in front of the participant (**e**).

The present protocol adapted an experimental setup described previously^43^. Participants stood with their backs facing a video camera placed 2 metres behind. The video, showing the participant’s body (virtual body) was projected in the body conditions in real time onto a head mounted display (HMD), see Fig. 4. While filming the video we also recorded the participant’s ECG throughout the entire experiment. Raw data (ECG) were acquired with the BioSemi Active II™ system (BioSemi, The Netherlands) at a sampling rate of 2048 Hz. In-house software (http://lnco.epfl.ch/expyvr) was developed to detect, in real time, the peak of each R-wave from the recorded ECG data and to trigger an additional visual stimulus (e.g. a flashing outline surrounding the participant’s virtual body) that flashed on and off synchronously or asynchronously with respect to the participant’s heartbeat (for further details please refer to Fig. 4). There were 2 different blocks corresponding to 2 different conditions: (1) Body with flashing outline synchronous with the heartbeat (body synchronous, BS); (2) Body with flashing outline asynchronous with the heartbeat (body asynchronous, BAS). Because of limited space in the faraday cage we were not able to test an object control condition.

#### Electroencephalogram (EEG) recordings and somatosensory evoked potentials (SEP)

Continuous EEG (BioSemi, Netherlands) was recorded at 2048 kHz sampling frequency from 64 scalp electrodes that were evenly distributed over the scalp according to the 10–20 EEG system. Electro-ocuolography (EOG) was also recorded. EEG epochs were calculated from 50 ms before to 100 ms after electrical stimulation of the right median nerve, which was delivered at 1 Hz (square wave pulses, duration 0.5 ms, mean intensity 12 mA, just below motor threshold). Epochs were averaged separately for each of the 2 experimental conditions (BS, BAS), baseline (BL) and for each participant to calculate the SEP. 400 epochs were recorded for each subject and each condition. Three subjects did not show the classical SEP components and were not included for the further SEP analysis. Using EEG and EOG channels, we used an automated artifact rejection based on signal amplitude and adapted to individual participant signal amplitude^71^. An average of 70 epochs (17%) were rejected per participant. In addition, all accepted trials were visually inspected at the level of traces, and scalp topography, to reject epochs with transient contaminating noise like eye blinks, eye movements, and muscle artifacts^46^. A 50 Hz notch filter was applied to reduce persistent noise. The data was bandpass-filtered between 30 Hz and 200 Hz and an average reference was applied. A baseline correction was calculated from −50 ms to 0 ms with respect to stimulus onset. Grand average SEPs across participants were calculated with SEPs normalized to their mean Global Field Power [GFP corresponds to the spatial standard deviation of the scalp electric field at a certain point in time^71^].

#### Procedure and experimental conditions

We performed the body synchronous (BS) and body asynchronous (BAS) conditions. We additionally recorded one block where the participants simply saw their body from the back but without any cardio-visual conflict [which we will refer to as the baseline (BL) condition].

After each block, participants were asked to complete a slightly adapted questionnaire from^43^ (question 1–8, see Supplemental Table 1), that allowed us to quantify self-identification with the virtual body. The questions were randomly ordered and rated on a 7-point Likert scale between −3 to 3, in which −3 indicated complete disagreement and +3 complete agreement.

An additional question assessed the intensity of the stimulation (Q9 “Please rate the intensity of the electric stimulation on a visual analogue scale between 0 (I didn’t perceive any stimulation at all) and 10 (This is the strongest pain I can imagine)). No participant reported pain or burning sensations.

### Statistical analysis

#### Behavioural data

Contrasts between BS and BAS conditions were carried out using paired t-tests, predicting higher ratings regarding self-identification with the virtual body in the BS condition as compared to the BAS condition. The significance level used (alpha) was adjusted using the Bonferroni method (p = 0.006).

#### SEP data

The grand average across BS and BAS conditions as well as BL at scalp electrode C3 [electrode over left somatosensory cortex contralateral to the stimulated right median nerve classically used for SEP analysis^46^] was computed in order to identify the N20 and the P45. Based on our hypothesis the N20 and the P45 were selected a priori for further analysis. The electrode ipsilateral to the stimulation (C4) was used as a control. Because of the inter-individual variability in SEP amplitude, values for each condition (BS, BAS) were calculated with respect to the BL^45^. This was done for each participant, each condition (BS, BAS) and the two components separately. These values were then subjected to statistical analysis [3-way ANOVA with factors electrode (C3, C4), synchrony (synchronous, asynchronous) and component (N20, P45)] and post-hoc analysis using the LSD test comparing the synchronous vs. asynchronous condition for the N20 and the P45 at each electrode. The significance level used was corrected for multiple comparisons using the Bonferroni method (p = 0.025). In order to further investigate the link between the modification of the P45 amplitude and the ownership with the virtual body, we correlated the amplitude of the SEP amplitude with Q3, using a permutation test (Spearman’s rank correlation coefficient analysis)^72^. In order to better compare the two distributions raw scores were transformed to standardized z-scores.

#### EEG Source localization

Neural generators for SEPs were computed applying a distributed linear inverse solution, based on a local autoregressive average [LAURA^73^], producing adequate source localizations with high temporal resolution^71^. Within the gray matter of the Montreal Neurological Institute (MNI) 152 template brain, we defined a solution space of 4022 evenly spread source points (or solution points). We transformed the MNI volume to a best-fitting sphere [SMAC model^74^] and used a three-shell spherical head model to calculate the lead field and the LAURA inverse solution. Anatomical labels are reported using an appropriate correction from Talairach–Tournoux to MNI space.

#### Data availability

The datasets generated during and analyzed during the current study are available from the corresponding author on reasonable request.

## Electronic supplementary material

Supplementary information
