# Supplementary material for: Cardio-visual full body illusion alters bodily self-consciousness and tactile processing in somatosensory cortex
Source: Sci Rep. 2018 Jun 18;8:9230. doi: 10.1038/s41598-018-27698-2 (PMC6006256; doi:10.1038/s41598-018-27698-2)
Supplement: Supplementary file 1 — Supplementary information [file 41598_2018_27698_MOESM1_ESM.pdf]

Supplementary online information

Cardio-visual full body illusion alters bodily self-consciousness and tactile processing in somatosensory cortex

Lukas Heydrich[1,2,ψ]*, Jane Elizabeth Aspell[1,4,ψ], Guillaume Marillier[1], Tom Lavanchy[1], Bruno Herbelin[1], Olaf Blanke[1,3]

ψ = contributed equally

[1]*Laboratory of Cognitive Neuroscience, Brain Mind Institute, Ecole Polytechnique Fédérale de Lausanne, 1015 Lausanne, Switzerland*

[2]*Department of Neurology, Inselspital, Bern University Hospital, University of Bern, Bern, Switzerland*

[3]*Center for Neuroprosthetics, School of Life Sciences, Ecole Polytechnique Fédérale de Lausanne, 1015 Lausanne, Switzerland*

[4]*Department of Psychology, Anglia Ruskin University, Cambridge, UK*

* Corresponding author:

Dr Lukas Heydrich Current address: *Department of Neurology, Inselspital, Bern University Hospital, University of Bern, Bern, Switzerland*

E-mail: lukas.heydrich@insel.ch

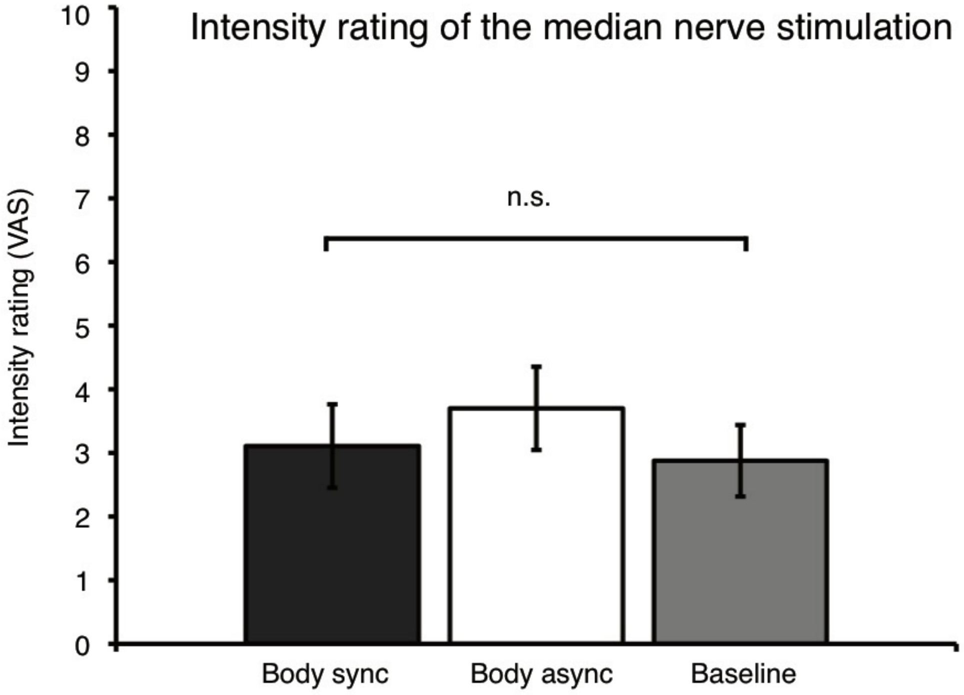

Supplemental Figure 1. Intensity rating. No significance difference was found for the intensity ratings between BS (3.1±2.3) and BAS (3.7±2.3), BS and BL (2.8±2), and BAS and BL (all $p>0.62$). Error bars indicate SEM.

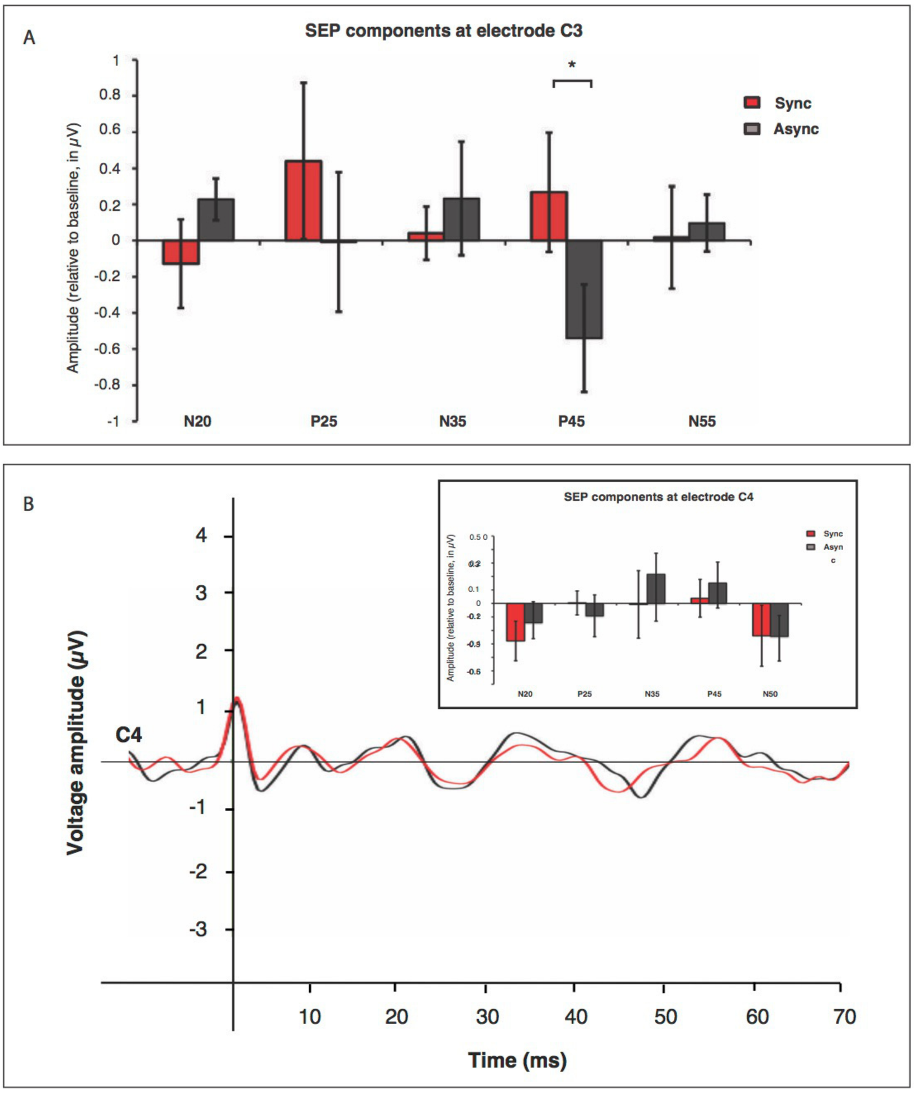

Supplemental Figure 2. A. Amplitudes relative to baseline for BS and BAS at electrode C3. We found a significant difference between BS and BAS only 45ms after electric right median nerve stimulation (P45). Red bars indicate amplitudes in the synchronous condition. Dark grey bars indicate amplitudes in the asynchronous condition. Asterisks indicate significant differences. Error bars indicate standard error of the mean (SEM). **B.** Amplitudes relative to baseline for BS and BAS at electrode C4 (control electrode). We found no significant difference between BS and BAS after electric right median nerve stimulation at similar latencies for any component. Red bars indicate amplitudes in the synchronous condition. Dark grey bars indicate amplitudes in the asynchronous condition. Error bars indicate standard error of the mean (SEM).

Table S1.

During the experiment there were times when :
1. It seemed as if I was feeling the electric stimulation where I saw the virtual body.
2. It seemed as though I was in two places at the same time.
3. I felt as if the virtual body was my body.
4. It seemed as if the electric stimulation I was feeling came from somewhere between my own body and the virtual body.
5. It felt as if my (real) body was drifting towards the front (towards the virtual body).
6. It appeared (visually) as if the virtual body was drifting backwards (towards my body).
7. It seemed as if I might have more than one body.
8. It seemed as if the flashing semi-transparent template was my heartbeat.

Supplemental Table 1. Questionnaire.

Component		*Latency (ms)*	*p-value*
**N20**	BS	19.82±1.49	0.52
	BAS	20.06±1.56	
**P25**	BS	25.48±1.85	0.47
	BAS	25.73±1.70	
**N35**	BS	35.98±2.53	0.26
	BAS	35.40±2.59	
**P45**	BS	45.75±3.31	0.29
	BAS	46.53±3.91	
**N55**	BS	57.03±2.44	0.75
	BAS	56.83±2.31	

Supplemental Table 2. No significance difference in terms of latencies was found between BS and BAS for any of the components at electrode C3.
